# Perspectives on automated composition of workflows in the life sciences

**DOI:** 10.12688/f1000research.54159.1

**Published:** 2021-09-07

**Authors:** Anna-Lena Lamprecht, Magnus Palmblad, Jon Ison, Veit Schwämmle, Mohammad Sadnan Al Manir, Ilkay Altintas, Christopher J. O. Baker, Ammar Ben Hadj Amor, Salvador Capella-Gutierrez, Paulos Charonyktakis, Michael R. Crusoe, Yolanda Gil, Carole Goble, Timothy J. Griffin, Paul Groth, Hans Ienasescu, Pratik Jagtap, Matúš Kalaš, Vedran Kasalica, Alireza Khanteymoori, Tobias Kuhn, Hailiang Mei, Hervé Ménager, Steffen Möller, Robin A. Richardson, Vincent Robert, Stian Soiland-Reyes, Robert Stevens, Szoke Szaniszlo, Suzan Verberne, Aswin Verhoeven, Katherine Wolstencroft

**Affiliations:** 1 Utrecht University, 3584 CS Utrecht, The Netherlands; 2 Leiden University Medical Center, 2333 ZA, Leiden, The Netherlands; 3 French Institute of Bioinformatics, 91057 Évry, France; 4 University of Southern Denmark, 5230 Odense M, Denmark; 5 University of Virginia, Charlottesville, VA, 22903, USA; 6 University of California San Diego, La Jolla, CA, 92093, USA; 7 University of New Brunswick, Saint John, E2L 4L5, Canada; 8 IPSNP Computing Inc., Saint John, E2L 4S6, Canada; 9 Westerdijk Institute, 3584 CT, Utrecht, The Netherlands; 10 Barcelona Supercomputing Center (BSC), 08034, Barcelona, Spain; 11 Gnosis Data Analysis PC, GR-700 13 Heraklion, Greece; 12 VU Amsterdam, 1081 HV Amsterdam, The Netherlands; 13 University of Southern California, Marina Del Rey, CA, 90292, USA; 14 Department of Computer Science, The University of Manchester, Manchester, M13 9PL, UK; 15 Department of Biochemistry, Molecular Biology and Biophysics, University of Minnesota, Minneapolis, MN, 55455, USA; 16 University of Amsterdam, 1090 GH Amsterdam, The Netherlands; 17 Technical University of Denmark, 2800 Kongens Lyngby, Denmark; 18 University of Bergen, 5020 Bergen, Norway; 19 Bioinformatics Group, University of Freiburg, 79110 Freiburg, Germany; 20 Sequencing Analysis Support Core, Leiden University Medical Center, 2333 ZC Leiden, The Netherlands; 21 Institut Pasteur, 75015 Paris, France; 22 IBIMA, Rostock University Medical Center, 18057 Rostock, Germany; 23 Netherlands eScience Center, 1098 XG Amsterdam, The Netherlands; 24 Informatics Institute, University of Amsterdam, 1090 GH Amsterdam, The Netherlands; 25 Leiden Institute of Advanced Computer Science, Leiden University, 2333 BE Leiden, The Netherlands

**Keywords:** scientific workflows, computational pipelines, automated workflow composition, semantic domain modelling, workflow benchmarking, bioinformatics, life sciences

## Abstract

Scientific data analyses often combine several computational tools in automated pipelines, or workflows. Thousands of such workflows have been used in the life sciences, though their composition has remained a cumbersome manual process due to a lack of standards for annotation, assembly, and implementation. Recent technological advances have returned the long-standing vision of automated workflow composition into focus.

This article summarizes a recent Lorentz Center workshop dedicated to automated composition of workflows in the life sciences. We survey previous initiatives to automate the composition process, and discuss the current state of the art and future perspectives. We start by drawing the “big picture” of the scientific workflow development life cycle, before surveying and discussing current methods, technologies and practices for semantic domain modelling, automation in workflow development, and workflow assessment. Finally, we derive a roadmap of individual and community-based actions to work toward the vision of automated workflow development in the forthcoming years.

A central outcome of the workshop is a general description of the workflow life cycle in six stages: 1) scientific question or hypothesis, 2) conceptual workflow, 3) abstract workflow, 4) concrete workflow, 5) production workflow, and 6) scientific results. The transitions between stages are facilitated by diverse tools and methods, usually incorporating domain knowledge in some form. Formal semantic domain modelling is hard and often a bottleneck for the application of semantic technologies. However, life science communities have made considerable progress here in recent years and are continuously improving, renewing interest in the application of semantic technologies for workflow exploration, composition and instantiation. Combined with systematic benchmarking with reference data and large-scale deployment of production-stage workflows, such technologies enable a more systematic process of workflow development than we know today. We believe that this can lead to more robust, reusable, and sustainable workflows in the future.

## Introduction

*Computational pipelines*, commonly referred to as scientific *workflow*s ^*^, play a key role in modern life science research. ^1– 3^ Analyses must be tailored to highly complex biological data by successive application of different algorithms and routines to maximize biological insight. Hence, scientists regularly use sophisticated workflows, composed from several software tools and data resources, for tailored data analysis processes. The highly dynamic eScience software ecosystem, which continuously sees new tools emerging, new reference data being provided and computational infrastructure improving, provides the basis for new and innovative workflows. Once developed, workflows are rarely considered stable, but are regularly adapted and reimplemented to meet the latest state of the art.

For more than two decades, dedicated scientific workflow management systems ^4– 9^ have been developed to support researchers at the different stages of the workflow development life cycle. ^10^ There is a flourishing ecosystem around these systems, including software-oriented ontologies, ^11– 16^ tool registries with rich metadata and functional annotations, ^12, 17– 19^ containerization technologies, ^20, 21^ workflow management and execution frameworks, ^7, 22– 25^ workflow repositories, ^26– 29^ workflow exchange formats, ^30^ and more. ^31^ Importantly, with the use of workflows in large scale data science and machine learning systems ^32– 36^ there has been a large increase in the interest in composing and executing workflows at scale. ^37^ These developments bring the long-standing vision of *automated workflow composition* ^38^ - the use of algorithms to perform the often tedious, time-consuming, limited and error-prone workflow development process - within reach.

To biologists there is a latent fear to have chosen the wrong computational paths for the analysis of their data, which could cause problems during the peer review, and in the worst case misdirect the data interpretation and invalidate downstream experiments. While human expert knowledge is an indispensable factor for validating and curating computational workflows, their automated assembly can significantly reduce the effort of getting from novel ideas to production and mainstream application, and at the same time help to increase scientific quality, reliability, and robustness. In fact, benefits of (partially) automated workflow development are manifold and include:

• *Minimal technicalities in software composition.* Manual workflow construction can be a tedious process. It requires the workflow developer to get familiar with the individual tools, sort out the compatibility of their input/output data formats, and connect them correctly to perform the intended process. An automated composer would not only save valuable research time, but also reduce errors.
• *Exhaustive exploration of data-analytical possibilities.* Given the abundance of bioinformatics tools available today, it is impossible for a human to consider all possible combinations that could be relevant for their problem. Indeed, scientists often resort to the tools and workflows with which they are familiar, at the risk of missing better suited or more effective pipelines for their problem. Assisted or even automated workflow composition would systematically and comprehensively explore the workflows that are possible with the available tools, and could also rank the possible workflows based on specific user requirements, such as runtime, compute requirements, underlying database usage, etc. This would enable new scientific findings by discovering well or better performing workflows that researchers would not have thought of themselves.
• *Generating ensembles of workflows.* When using workflows to test biological hypotheses, automated workflow composition enables us to generate ensembles of orthogonal workflows combining different tools and services seizing on different aspects of the data (for example, algorithms that concentrate on different subsets of the raw data). This idea has been proposed by Gil *et al*. ^39^ and is not epistemologically novel. As Hempel summarized over half a century ago, “The confirmation of a hypothesis depends not only on the quantity of the favorable evidence available, but also on its variety: the greater the variety, the stronger the resulting support”. ^40^ As a single, linear, workflow is typically unable to collect *all* available evidence and parallelization is not always an option, workflow ensembles can provide additional confidence in rejecting null hypotheses.
• *Repairing workflows by tool substitution.* Within a strictly and semantically well-defined context, alternative, semantically equivalent tools or services may be fully automatically substituted when the default is deprecated or unavailable. In a less well-defined setting, the workflow developer might still be semantically guided towards possible alternatives and receive suggestions for sensible replacement tools. Ideally, the resulting workflows would also be tested automatically, to check if they produce the same or similar output as the old workflow on available benchmarking data. Workflows that can be automatically repaired in this way are inherently more robust and viable.
• *Optimizing workflow output.* Workflow topology, components as well as parameters can be optimized in an integrated workflow composition and benchmarking framework. This can be used, for example, to maximize output, e.g. identified proteins in a proteomics experiment, or minimize some computational resource, e.g. memory or CPU time. Specific properties of a data set might influence such optimization adapting the methods not only to the data type but also the data itself.
• *Ensuring the methodological quality of workflows.* Automated composition can ensure that data is correctly used within components (e.g. training and test data are properly used in machine learning). Likewise, it can prevent errors in parameter setting as well as combinations of components.

In this article we report on the state of the art of automated workflow development in the life sciences, discuss current and future challenges and develop perspectives for the coming years. The report is based on discussions during a Lorentz Center workshop (held at the Lorentz Center in Leiden, Netherlands, from 9-13 March 2020) dedicated to this topic ^41^ (workshop program available in *Extended data* ^115^), with the authors as participants. In the section Workflow life cycle we outline a “big picture” of the scientific workflow development life cycle, before surveying and discussing current methods, technologies and practices for semantic domain modelling (section Semantic domain modelling), automation in workflow development (section Automation in workflow development), and workflow assessment (section Workflow assessment). In the Roadmap section, we derive a roadmap of individual and community-based actions to work toward the vision of automated workflow development in the forthcoming years. Finally, the Conclusion section wraps up the discussion.

## Workflow life cycle

**Figure 1.**
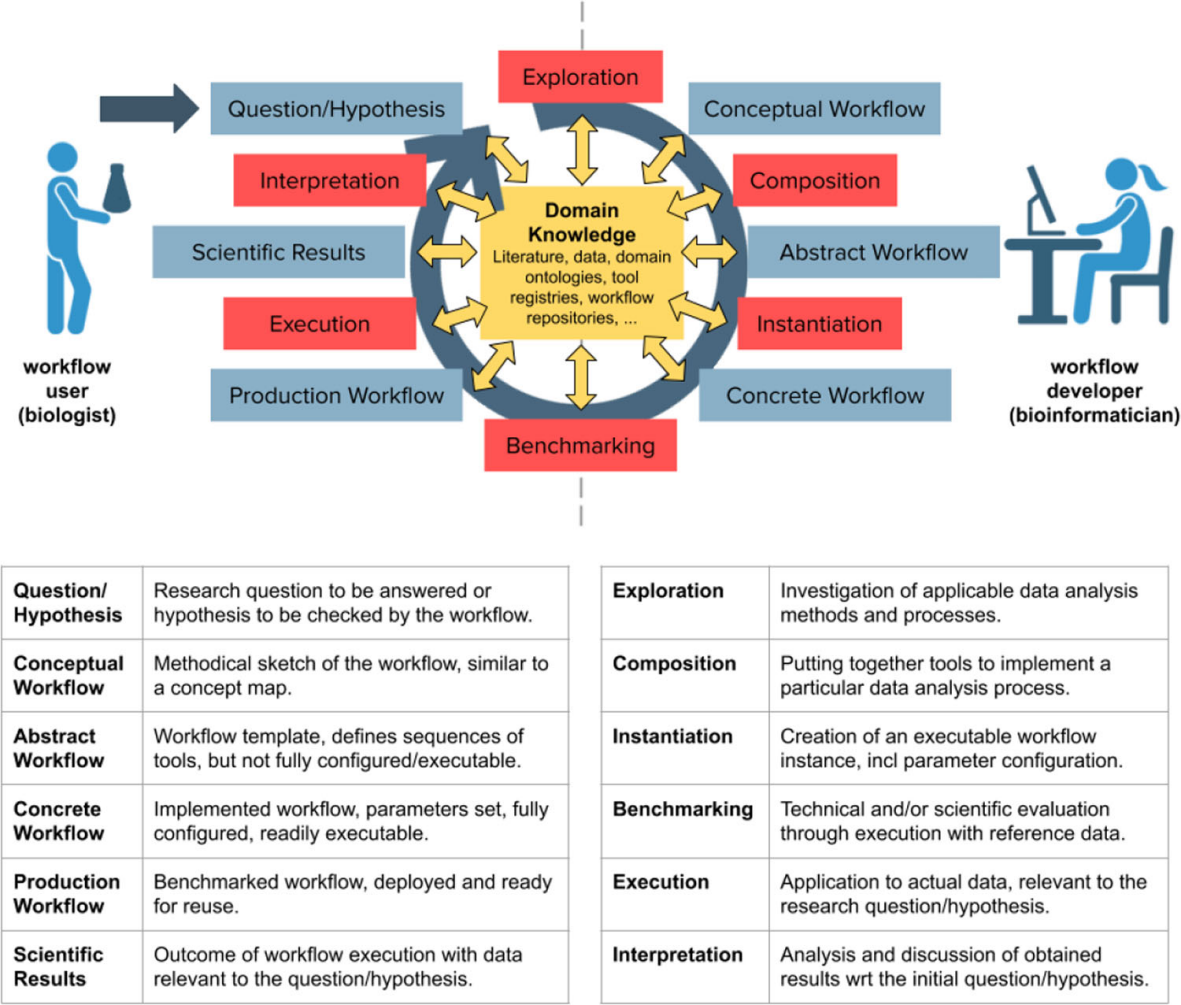
Scientific workflow life cycle.

The development of scientific workflows is an involved, multistep, and often iterative process. The schematic process in Figure 1 captures the “big picture” that emerged from the discussions at the Lorentz Center workshop. It extends earlier descriptions of the scientific workflow life cycle, ^42– 44^ and will provide guidance for the discussion of automation approaches in the remainder of this article. The life cycle distinguishes six principal stages:

1. The *scientific question* to answer, or the *hypothesis* to test. It guides the subsequent exploration of suitable analysis methods, as well as for the choice of data, methods, tools, platforms, and interpretation of results.
2. The *conceptual workflow*, as a sketch of the methodical steps that the workflow should perform on data from a specific experiment type, from a domain-specific perspective. It is the result of exploring possible analysis methods for the scientific question/hypothesis and the data at hand. It can be formalized, for example as a Concept Map, ^45, 46^ but often it will only take the form of a paper or mental sketch. Nevertheless, it is an important stage in the workflow development process.
3. The *abstract workflow*, describing sequences of computational tools that implement the conceptual workflow. It is the result of composing individual tools into workflows, taking into account the compatibility of their input/output types and other kinds of static information. An abstract workflow is not yet (fully) configured, however, and thus not readily executable.
4. The *concrete workflow*, as the fully implemented, fully configured and readily executable stage. It is the result of instantiating an abstract workflow with the relevant data and parameters.
5. The *production workflow*, deployed and ready for (re) use by other parties. It is the result of benchmarking different variations of a workflow in order to arrive at a tested and robust version for wider use.
6. Finally, the *scientific results* that emerge from executing the workflow with the research data. They are interpreted by the domain scientists, and ideally shared with others in a manner that promotes reproducibility and transparency. This often leads to new scientific questions or hypotheses, to be addressed by another workflow.

In practice, these stages are often not so clearly distinguishable. They can be interleaved, skipped, and taken in a different order than the life cycle suggests. A non-exhaustive list of examples includes:

• A workflow developer might not produce an (explicit) conceptual sketch of the workflow before starting to explore and compose tools, but rather do so in one go.
• Trying to compose an abstract workflow might reveal that the research question/hypothesis and/or the conceptual workflow need to be refined.
• Many popular workflow management systems, such as Galaxy, ^7, 57^ handle both composition and instantiation simultaneously and combine abstract and concrete workflows in one formalism. Typically, they also allow for workflow execution for both benchmarking and production purposes, thus covering additional stages in the life cycle.
• A benchmarked workflow might be used to generate results, but is never actually deployed for reuse by others.
• Existing workflows from repositories like myExperiment, ^28, 29^ Dockstore ^47^ or the WorkflowHub ^26^ can be reused at different stages, preceding stages in the principal life cycle to be either skipped or shortened.
• Popular production workflows, such as those provided by the Bioinformatics Core Facility ^48^ are routinely used by researchers in a close execution -> results -> interpretation -> execution sub-cycle.
• Specific data and study properties pre-determine workflow components by prior knowledge about tool performance.

The figure also indicates the importance of literature, data, domain ontologies and tool registries and workflow repositories. They provide the basis for exploring, composing, implementing, running, evaluating, sharing, and reusing computational pipelines, and are thus central to the whole workflow life cycle. In fact, they are the enablers of many of the “shortcuts” outlined above.

Finally, the figure distinguishes two principal roles in the workflow life cycle: 1) the workflow user, here represented by a wet-lab biologist, who has research questions and data for which they use computational tools and workflows to obtain results, and 2) the workflow developers, here represented by a technology-oriented bioinformatician, who has the skills to develop and provide computational workflows for their colleague’s data analysis problems. While there are obviously individuals who perform both roles, there is an increasing specialization happening in the field of scientific workflows, with research software engineers skilled in workflow technologies emerging as a professional profile in its own right. ^49^

## Semantic domain modelling

In the context of scientific workflow development, the semantic domain model is (formalized) knowledge about the technical entities within a domain. It includes domain ontologies as controlled vocabularies for annotating entities with metadata, and registries and repositories of annotated data, tools and workflows. For the purpose of automating (parts of) the workflow construction process, tools and their functional annotations are of particular importance. Possible connections of individual tools are in the first place determined based on the annotated input/output data types and formats.

The eScience community, and especially the life science circles, were early adopters of semantic technologies. For example, driven by the myGrid project in the UK, the myGrid Ontology ^16^ was an early initiative of a software-oriented ontology designed to facilitate bioinformatics service discovery, and the BioCatalogue ^18, 19^ was one of the first domain-specific web service registries, providing a curated collection of semantically annotated bioinformatics services. Around the same time in the same context, myExperiment ^28, 29^ emerged as one of the first repositories for scientific workflows, allowing users to upload, describe, annotate and share their computational pipelines. As a successor to myExperiment, EOSC-Life has now established the FAIR Computational Workflow registry WorkflowHub. ^50^ Whereas myExperiment treated workflows as data objects, WorkflowHub recognises them as software objects with dependencies and other properties.

Over the last decade, these early ideas, approaches and platforms have evolved further, and are now increasingly being adopted by the life science and wider eScience communities.

### Examples of semantic domain models

Three important, contemporary and active semantic domain modelling platforms are EDAM/bio.tools, OntoSoft and SADI. They support the production and dissemination of semantic software descriptions that help to make these tools more FAIR (Findable, Accessible, Interoperable and Reusable). ^51– 53^

*EDAM and bio.tools*

The EDAM ontology of bioinformatics terms ^14^ and the bio.tools registry ^54, 17^ have become the primary resources for semantic software annotation in the European life sciences community. EDAM provides a controlled vocabulary for the annotation of computational tools with relevant bioinformatics topics, performed operations, as well as type and format of the input and output data. The bio.tools registry uses EDAM for the fine-grained semantic description of tools and their functionality according to a pragmatic model defined in the biotoolsSchema. ^55^ The annotations facilitate the discovery of individual tools, and the assessment of their (inter) operability such as their combination into workflows. The development of both EDAM and bio.tools is driven and supported by the broader community.

bio.tools is part of the ELIXIR Tools Platform and becomes increasingly connected with its other services such as BioContainers, ^56^ Galaxy, ^7, 57^ BioConda, ^58^ WorkflowHub ^26^ and OpenEBench, ^59^ as well as external services like Debian Med. ^60^ This will form a centralised, transparent ecosystem of information about tools and services in the life sciences. Here, EDAM serves as a common language to connect and enrich extensive software dossiers.

*OntoSoft*

The OntoSoft ontology ^20, 21^ has been designed as an ontology for scientific software metadata. OntoSoft allows for the description of software. This includes understanding how to access and update that software, how to execute it, how to use it, and information on who supports the software. The OntoSoft ontology is the basis for the design of the user interface in the OntoSoft portal, the organization of the underlying knowledge base, and the integration with other software repositories. Although OntoSoft is currently focused on earth sciences applications, providing geoscientists in the NSF EarthCube project ^61^ with an intelligent system to share and reuse code, its principles are equally applicable in other domains.

OntoSoft-VFF (Ontology for Software Version, Function and Functionality) ^62^ extends OntoSoft. It stores semantic software metadata needed to manage workflow evolution and updates, suitable to help scientists to find and select the right tools to implement given workflow steps, explore alternative tools to use in their workflows, and keep track of tool and workflow changes. Similarly, OntoSoft is the basis for OKG-Soft, ^63^ an open knowledge graph that describes scientific software in a machine-readable manner and supports the FAIR principles for software.

*SADI registries*

SADI (Semantic Automated Discovery and Integration) ^64^ is a framework for creating Semantic Web Services and a design pattern for the formal description of the service interfaces. Services are described by an ontology that defines I/O class names, predicates and service names with a unique URL. The ontology specifies an explicit relationship (semantic predicate) describing the functionality of a service between the I/O, for example “getDrugNamebyDocument”. ^65^ The service descriptions are collected in a SADI registry. From there, SADI Services can be readily discovered and composed into workflows, as all services consume and generate RDF (syntactic interoperability) and thus the output of one SADI service can be directly consumed by any other SADI service. Through the provisioning of Semantic Web services on top of relational databases for semantic querying, SADI facilitates both data-as-a-service and algorithms-as-a-service. Recently Valet SADI ^66^ was developed as a service generator for assisting the technically involved authoring of SADI Web Services. Designed as middleware, SADI is not accessed directly, but through specialized query engines (see section SHARE & HYDRA).

### Discussion of semantic domain modelling

Semantic domain modelling is hard. ^67^ Especially in highly collaborative community efforts like EDAM/bio.tools, OntoSoft and SADI, it is important to realize that the controlled vocabulary defined by the domain ontology constitutes a kind of social contract that all tool annotators must understand and respect. Using the same interpretations of the terms defined by the ontology is crucial for the meaningfulness and consistency of the domain model.

To be useful for practical application, ontologies have to be designed for a clear purpose. In the context of workflow composition, it needs to be defined, for example, if the ontology is supposed to help the (manual) search for and/or the automated composition of computational tools, and if it targets the creation of informatically, bioinformatically and/or biologically valid workflows. Furthermore, the ontology needs to use an adequate level of detail, neither too simple nor too complex, to avoid overgeneralization as well as overfitting. These challenges are both technological and social, with the latter typically being harder to address. This was also reflected by the discussion of semantic domain modelling during the Lorentz workshop, with the use of EDAM and bio.tools as guiding examples.

*Scope*

In the case of bio.tools, the EDAM ontology and the biotoolsSchema provide a technical basis and general direction for the annotation of bioinformatics tools in the registry. However, they leave room for interpretation, calling for clarification. What kinds of tools are in scope, and what exactly should be included in their annotation?

*Content*: The bio.tools Curators Guide ^46^ defines the scope of relevant tools as “application software with well-defined data processing functions (inputs, outputs and operations)”. This clearly includes, for example, command-line tools for sequence alignment, or web services for database searches. For other workflow building blocks this is less clear. On the one hand, workflows often require the inclusion of “shims”, ^68, 69^ small formatting or conversion tasks between the actual data processing steps. They are often not considered tools in their own right, but are indispensable for interoperability. Automated workflow composers as well as human workflow developers would hence benefit from their availability in the registry, and it would prevent a lot of reinvented wheels. While these are strong arguments for the inclusion of shims in bio.tools, there is also a certain risk of fragmentation and overloading the registry with trivial functionality that needs to be managed. On the other hand, workflows can also be considered tools that can be used in (other) workflows. From the perspective of a tools registry it is desirable to include them as “black boxes” providing certain functionality as a service. The inner workings can be visible in a workflow repository like the WorkflowHub, similar to the source code of other computational tools being available in a repository like GitHub.

Similarly, clear guidelines are needed for meaningful annotation of tool suites and multifunctional tools. The bio.tools Curators Guide recommends registering tool suites as such (the biotoolsSchema foresees a tool type “Suite”), but to also add separate entries for the individual tools to capture their functionality clearly. An example is the SAMtools suite ^70^ and its members, such as samtools_sort and samtools_slice_bam. Multimodal tools with different functions should be annotated with multiple specific functions (as supported by the biotoolsSchema) rather than trying to cover all modes of operations in one generic annotation.

*Annotation*: The biotoolsSchema includes various kinds of attributes that can be used for tool annotation. The bio.tools Curators Guide ^46^ provides guidance for tool annotators and describes which information must, should, should not, and must not be included. For automated workflow composition, the annotation of tool function (performed operation, data type and format of inputs and outputs, execution command) is essential. In practice annotators often face ambiguous situations, such as:

• Tool inputs can be distinguished into payload data and configuration parameters. For example, a set of sequences to be aligned is payload data, and a gap penalty value is a parameter for an alignment tool. This distinction is however not always clear. For example, a substitution matrix used by the algorithm as a parameter but provided as an additional input file could be placed in either of the categories. Currently tool annotation in bio.tools mostly focuses on the annotation of payload input (for pragmatic reasons such as conciseness and limitation of complexity), but for full automated workflow construction information about the parameters to be configured would also be required.
• The biotoolsSchema readily distinguishes between the type (kind of content, domain perspective) and the format (representation, technical perspective) of input and output data. For example, an “RNA sequence” is a type, and “FASTA” is one of the possible formats in which it can be represented. This distinction is however not always easy to make. The IUPAC International Chemical Identifier (InChI), ^71^ for example, is classified as a format in EDAM, but it could also be viewed as a type of data. The domain ontology should have clear criteria for the classification of such formats, and ideally make sure that all formats included are connected to at least one type of data (and vice versa) to enable meaningful tool annotation.
• Composite data formats contain different parts, where a specific tool might only use one or some of them. For example, RetroPath 2.0 ^72^ works with InChI identifiers that are available from one column of a CSV file. Currently it is not clear how these would be modeled in the ontology and annotated in the registry in the best way. Possible solutions might include a combination of reusing approaches such as the structured metadata for CSV and other tabular data ^73^ and providing corresponding, ideally automatically generated, shim libraries.

Clearly, the domain ontology needs to provide a vocabulary that supports the required annotations and the desired level of granularity. As the needs change, also the ontology has to evolve. EDAM is indeed continually evolving based on input from the bioinformatics and, in particular, the bio.tools community. It is for example well developed for the proteomics domain, due to recent work on (automated) workflow composition and benchmarking.

*Quality*

The quality of automatically composed workflows critically depends on the quality of the domain ontology and tool annotations (“garbage in, garbage out”). ^74, 75^ Hence it is important that all tool annotations consistently adhere to the curation guidelines that capture the defined scope and annotation conventions. Consequently, if annotations are too vague or imprecise, automatic composition will likely generate incorrect or nonsensical workflows. If they are overly specific or narrow, possible workflows might be overlooked due to overfitting. This sounds simple, but is difficult in practice. It requires not only a thorough understanding of the curation guidelines and annotation conventions, but also expert-level knowledge in the application domain of the tools, and ideally practical experience of using them. Variations in the stringency of rules for the annotation being followed have direct effects on the interpretability of queries to the system.

Note that the semantics of the annotation is typically limited to a positive tagging, that is, no negative statements as in “not performing indexing” can be expressed, and there are no set operations like intersections or exclusions. When also allowing negative statements, or exceptions to a universal quantifier, this easily leads to semantically incorrect workflows merely by omission. Thus, negative statements or set operations shift responsibilities for correctness to the maintainers of higher-level resources like ontologies. This poses a challenge to synchronize the development of catalogs and ontologies as their granularity in biological expressiveness needs to match the decision making of users for the applicability of scientific tools for a given problem. With a larger amount of scrutiny on the ontologies than on individual catalog entries, this may support stringency and help overall quality.

The quality problem is aggravated when there is a need to annotate large numbers of tools (at the time of writing, for example, bio.tools comprises almost 19,000 entries). Ideas to scale up annotation rates include text-mining of annotation information from the tool’s documentation pages, deriving annotations from other repositories (such as the Galaxy Toolshed ^76^), and the automation of semantic description of tools and services via propagation from tried-and-tested workflows. ^77^ While enabling the inclusion of more tools in a shorter time, such automated approaches do not guarantee a consistent high-quality annotation of tools. Therefore, automatically annotated tools should be checked for a minimal level of curation quality before being made available for automated workflow composition.

A community registry like bio.tools needs to find a balance between being open to contributions and curating entries for quality control. At the end of the day the (manual) curation work invested needs to reflect the actual usage, i.e. frequently used tools justify greater curation efforts. Curators need adequate training to become able to deliver high-quality annotations. While desirable, it is unrealistic to assume that this is feasible to provide to everybody who might (potentially) contribute. A pragmatic way out might be to mark curated entries to distinguish them from unchecked ones, for instance with a tag or badge denoting that the functional tool annotation was checked and the tool is suitable to be used by automated workflow composers. This could be combined with procedures to develop consensus annotations for widely used tools by a group of experts, which can then serve as landmark or even “gold standard” examples for tool annotation. Furthermore, technical monitoring of tools, continuously performed by e.g. OpenEBench, ^59^ could provide up-to-date information about tool status and availability. In any case these mechanisms should be defined in a governance model, together with the processes for maintaining and updating the entries in the registry.

Finally, it is not only tools that develop, but also their metadata in the registries. Inconsistencies can easily lead to errors or unsatisfactory performance of automatically composed workflows. Manual verification is likely to fall short, especially since registries take a passive stance towards tools’ updates. With good reference workflows and benchmarking data available, however, workflows could be tested automatically as a joint quality control for the tools and their semantic descriptions in the registry. One may anticipate that such an automated quality assurance even prepares for workflow optimization.

*Incentives*

The success of registries like bio.tools depends on community contributions, which is predicated upon the motivation of tool users and developers. There are several incentives for potential contributors. For example, for tool developers, rich annotation of their software will increase its findability and comprehension, and thus its potential to be (re-)used individually or within a workflow. This in turn can improve the impact and eventual citations of the software. Improved tooling (intuitive user interface, annotation help, very-well defined metadata schemas) that integrates well with the tool development infrastructure can help to lower the threshold to tool registration and annotation. Registering tools in a community is also in line with recent practice guides like the “Four simple recommendations to encourage best practices in research software” ^78^ and the “Five Recommendations for FAIR Research Software”, ^53^ which are increasingly attracting the attention of organizations and project-funding agencies. Similarly, publishers might require the registration of tools in a community registry as a condition to accept papers that are describing or using the software, providing additional enforcement. A related problem is the incentives for updating registry entries when the respected tool has changed (updates, new versions, new features).

Ideally, the problem would be solved through “knowledge acquisition by stealth”: sneaking in metadata curation steps in people’s normal routines, and e.g. scrape them from available documentation or with smart tools that integrally capture the semantics from the start, so that people do not feel like having to do something extra. This is an appealing but complex long-term goal, however, which requires consideration of the entire lifecycle of tool and workflow development.

## Automation in workflow development

The possibilities for automation in the multi-stage workflow development process are manifold and range from assistance in specific phases to full automation. The most likely basic form of assistance, the possibility to search for available components with keywords or filter criteria, is a common feature of visual and interactive workflow management systems. More sophisticated is the assistance through context-dependent suggestions, which can take the form of guided workflow construction. An early example here is the ontology-driven assisted web service composition facilitated by BioMoby, ^79^ which was integrated in the Taverna workflow system to guide workflow construction. ^80^ Further down in the workflow life cycle, automated service configuration can assist the user to set parameters and get the workflow ready for execution. Automated service substitution aims to replace unavailable or otherwise deprecated tools by semantically equivalent but operating ones, to repair a workflow and make it executable again. Related, automated shim suggestion is intended to introduce mere technical steps (such as reformattings or format conversions) between the actual data analysis tools. One of the most intriguing forms of automation in workflow development is the automated anticipation, or exploration, of entire new workflows. Systems like Magallanes ^81, 82^ and PROPHETS, ^83, 84^ for example, have already demonstrated about a decade ago that AI planning and program synthesis techniques can be applied in pursuit of this goal.

### Examples of approaches to automation in workflow development

At the Lorentz workshop, the following four current and active approaches to automation in the workflow design process were presented and discussed in greater detail.

*The tool recommender system in Galaxy*

Galaxy ^7, 57^ is a popular web-based platform for high-throughput sequencing data and other big data analyses. Researchers can use it to share data and analyze them by running workflows. Workflows can be imported (shared workflows), extracted from history, or built manually. With more than 2,000 tools available in Galaxy, users need guidance during workflow construction. Therefore, a recommendation system has been developed to suggest possible next tools in a workflow under construction. ^85^ The recommender system uses an approach based on deep learning. The idea is to learn possible tool combinations from existing workflows, and use this knowledge to suggest tools for new workflows. The model is trained on tool sequences that are extracted from workflows. A Recurrent Neural Network with gated recurrent units (RNN-GRU) ^86^ is used, with tool usage as weights. The recommender system uses the trained model to predict possible next tools, ranking them by a score that is provided by the model and indicates the prevalence of the respective combination.

*Workflow INstance Generation and Selection (WINGS)*

WINGS ^6, 39, 87^ is a semantic workflow system that assists scientists with the design of computational experiments. A unique feature of WINGS is its high-level semantic workflow representations that are automatically configured and customized into executable workflow instances. Therefore, WINGS employs workflow reasoning algorithms that use constraint-based planning. The constraints can reference both workflow constituents (steps, data, parameters) and metadata of input datasets, and are used to customize a workflow to a given dataset. For example, a constraint could require that the alignment step and the assembly step in a bioinformatics pipeline are done with the same reference genome. Such constraints capture domain expertise about workflows (as purpose-specific combinations of tools) that goes beyond what the metadata of the individual tools can express.

Another interesting feature of WINGS with regard to automation is that it allows for the use of abstract (in terms of our Figure 1: conceptual) steps in the workflow, which can be implemented by different tools or sub-workflows. For example, PeptideSearch is a method that is performed by the tools X! Tandem, ^112^ MSGF+ ^113^ and Myrimatch. ^114^ The implementations can flexibly be chosen and exchanged, making it easy to quickly generate and compare workflow variants, for example to assess the robustness of the method or to take part in benchmarking challenges. ^88^

*The Automated Pipeline Explorer (APE)*

APE ^89^ is a command-line tool and application programming interface (API) for the exploration of possible workflows in large collections of semantically annotated tools. Input for APE is a high-level workflow specification that captures the user’s intents. It includes the available input data (type and format), the desired output data (type and format), and possibly additional constraints (such as tools to use or to avoid). For example, a proteomics workflow might be specified (using EDAM terms) as taking “ Mass spectrum ” in “ Thermo RAW ” format as input, producing an “ Amino acid index ” in any format (see here ), and using a “ Retention time prediction ” operation in the analysis. When applied to bio.tools, APE finds several workflows that meet this specification.

The exploration algorithm in APE is a variant of LTL-guided program synthesis, implemented as bounded search through iterative deepening. ^90, 91^ The domain model (ontology and tool annotations as provided by EDAM and bio.tools) and workflow specification are encoded as a SAT instance (a propositional Boolean formula in conjunctive normal form), and given to a SAT solver to find a satisfying assignment of variables. The SAT solutions are translated back into actual workflows, which can be transformed further into, for example, executable shell scripts, CWL workflows or other representations.

*SHARE & HYDRA*

SHARE ^92^ and HYDRA ^93– 95^ are specialized query engines to work with SADI registries. They receive user input in the form of SPARQL queries and use the registry as a knowledge base for automated workflow composition, matching the query to thousands of services (Data as a Service (DaaS)/Application as a Service (AaaS)). Concretely, a SADI query engine maps triple patterns from the WHERE clause of a SPARQL query to indexed SADI properties in the registry. In particular it checks the I/O descriptions to ensure compatibility between services. In doing so it discovers SADI Web Services capable (when combined in a workflow) of generating the required triples. Finally the query engine plans and orchestrates a workflow with calls to RESTful web services, integrating service outputs locally in RDF.

While SHARE supports the construction of the queries through a textual mechanism called SPARQL Assist, HYDRA also provides a keyword-based and graphical interface. Moreover, HYDRA performs reasoning on an input SPARQL query with respect to ontologies, leveraging the service registry to identify service calls may help answering the query. Iteratively, data returned from service calls triggers new registry calls to identify further relevant service calls. Through this incremental workflow extension, answers are produced incrementally with the user in the loop. The process terminates when all service calls have been made and all available answers have been produced.

### Discussion of approaches to automation in workflow development

The four approaches sketched above introduce automation to different phases of the workflow development life cycle, thus complementing each other. In the following we discuss cross-cutting aspects of automation in different phases of the life cycle.

*Target audience*

The different approaches clearly fit different user profiles and are intended to serve different target audiences. It seems useful to broadly distinguish between biologists as workflow users and bioinformaticians as workflow developers (obviously there are people who qualify for both roles). Many biologists simply want answers to their (biological) questions. They want to be able to find software solutions for their computational problems that they can trust, that have been tested and evaluated, that are reliable and that will run. They are not interested in the technical details of tools, workflows or even their construction processes. Despite the increasing integration of (bio) informatics education in life science curricula, this is not likely to change much. Hence this group of users is a target audience for production workflows as end results of the workflow development process. The development of workflows is in the hands of tech-savvy bioinformaticians. Within this large group, it seems that the “workflow engineer” emerges as its own professional profile. They compose, curate, evaluate and deploy workflows for specific bioinformatics problems, and make them ready for the actual end users.

Of the automation approaches sketched above, HYDRA is the only one that directly and explicitly targets a workflow end user. This is made possible by the careful service curation that enables the direct execution of the workflows composed for the queries. The other approaches are better suited to support workflow developers in different phases of the construction process. APE is most suited to supporting workflow exploration and composition in an early phase, acting as a “route planner” that automatically explores and generates new possibilities of workflows for an abstractly described problem. The obtained “recipes” can then be developed further into concrete and executable workflows. The Galaxy tool recommender also targets the early, still exploratory phases of workflow construction, but based on a concrete workflow under construction. WINGS takes an abstract/template workflow as the basis, and then takes care of automatically instantiating it to obtain a concrete and executable workflow. Interestingly, to the best of our knowledge, the existing approaches do not cover automated workflow benchmarking yet, which will however be essential for bringing automatically created workflows to the production stage.

*Applicability and trust*

All approaches have in common that their applicability depends on the quality of the underlying knowledge base or semantic domain model (cf. Section Semantic domain modelling). Generally, a somewhat lower quality seems to be tolerable for assisted workflow composition, as the developer can correct or discard suggestions based on their domain knowledge. This is the case, for example, when using a tool recommender system, like that in Galaxy, where the user can at any point decide whether or not to follow the recommendation. Semi-automated approaches like in APE and WINGS require higher-quality semantic annotations, but as the workflow developer still has the possibility to check and revise the workflow before execution, they can tolerate medium-quality annotations to some extent, Complete automation is possible for specific application areas or use cases with well-defined domain knowledge and high-quality annotations. As the required curation efforts are substantial, it is not realistic to achieve this in a generic framework. HYDRA instances, for example, are set up for well-defined application areas and invest in a rigorous semantic annotation of the provided Web Services. As a result, end users can use HYDRA to query for and directly execute workflows. Another good example for complete automation in a well-defined scope is Automated Machine Learning (AutoML), ^35^ where machine learning models are generated automatically given a start and end point.

Conversely, the higher the degree of automation, the more the user needs to decide whether to blindly trust the outcome, or conduct checks to verify its plausibility and correctness. This is again connected to the quality of the knowledge base/semantic domain model, but also has to do with the degree of involved “blackbox” behaviour. For example, a HYDRA workflow is not easily validated before execution (as it is directly executed during construction), but the recorded provenance data make it possible to inspect and assess the performed computations afterwards. In contrast, the Galaxy tool recommender leaves the control about selecting workflow steps to the user, but requires trust in the quality of its recommendations. As they are machine-learned from tool combinations in existing workflows, there is likely a bias towards frequently used and against less used and new tools. Further metrics and criteria to base recommendations on are of course possible (such as a functional similarity index, compatibility, citation index or novelty), but in any case they should be made transparent to the user and create awareness for possible biases.

*Abstract Workflows*

Different levels of abstraction are key to differentiating the phases of the workflow development life cycle and assess the potential for automation ( Figure 1). Indeed, automation in the development process usually means automated concretization. The process starts with a domain-level problem description as an initial idea, and then phase by phase boils it down to a concrete implementation, until a production workflow brings it back to the domain level and end user.

The term *abstract workflow* has been used with different meanings. ^38^ In the executable Common Workflow Language (CWL), ^30^ for example, it is possible to define a workflow with step/tool implementations (e.g. Docker containers) or with abstract placeholder operations; in either case the workflow defines and connects all workflow steps and their parameters. In CWL an abstract workflow can thus be classified as containing one or more placeholder operations; note however that CWL engines may still permit partial workflow execution for the concrete steps.

In CWL such abstract operations can still refer to a concrete tool that should be used when implementing the workflow for execution (omitting details such as command line), or to a class of possible tools (e.g. by using an EDAM operation term). In WorkflowHub, CWL is used as the canonical workflow description for workflows where possible, alongside the native workflow description: for example a Galaxy workflow for Climate analysis that is expressed in Abstract CWL. ^96^ In WINGS, an abstract workflow is even more generic and flexible, allowing not only for abstract operations, but also for missing steps that are filled in during workflow elaboration.

Here we separate the notions of *conceptual* and *abstract* workflows (cf. Fig. 1): We define a **conceptual workflow** to be a sketch of a data analysis pipeline, similar to a concept map, ^45^^,^^46^ that describes the process in domain-level but implementation-independent terms, for instance using operation and data terms from the EDAM ontology. Next to that, we define an **abstract workflow** to be a template that describes complete sequences of computational tools, but that is not yet fully configured and executable. Such conceptual and abstract workflows make it possible to focus on the workflow steps without complexities such as parameter settings, execution or data sets. They are also convenient for enabling search (both for users and automation) and comparisons with other workflows, thus providing a suitable exchange level and intersection point for different stakeholders and communities. They can be the target (e.g. the result of workflow exploration with APE) as well as the input (e.g. a starting template for workflow elaboration with WINGS) for automated composition approaches.

Furthermore, abstract workflows can be obtained by automated abstraction from (collections of) concrete workflow instances. ^97^^-^^99^ They are useful for better describing, understanding and evaluating workflows, and for preserving the essence of computational pipelines in automatically generated method sections also when the executable instances of the workflow decay. ^98^ They can therefore provide a way to document workflows in a FAIR way. ^50^^-^^52^

## Workflow assessment

The abundance of computational tools available in today’s eScience ecosystem leads to an enormous number of possible workflows. With automated composition approaches these can be more easily accessed and generated. There is a problem of identifying and selecting the “best” (whatever that means in the concrete case and context) alternatives among various options at different stages of the workflow development process: Which methods should be chosen given the available data and the analysis goals? Which individual tools should be given preference over others? Which combinations of tools to favor over others? Which workflow candidates to select for implementation? Which workflows to bring into productive use?

To enable automated composers to make informed choices, tools and workflows need to be compared with well-defined meaningful criteria. To structure the discussion of possible criteria and selection strategies, we follow the traditional distinction between static (based on information that is available without execution of the workflow) and dynamic (based on workflow execution) analysis.

### Static analysis

Static analysis is performed on the “source code” of the workflow (may be in a classical scripting language or a specific workflow formalism) without actually executing it, treating the individual tools as black-box building blocks that have particular (static) properties. As such, static analysis mostly concerns the early stages of the workflow development life cycle, where the workflow developer (human or machine) can use available information about individual tools and tool combinations to explore possible workflows and the tools to implement them. Arguably, static analysis is often less meaningful than the results obtained through actual execution, but provides a powerful way for pre-screening the capability of a workflow. In addition, in many cases it is simply not possible to implement and execute all possible workflows in order to choose between options. Hence it is important to leverage what can be said about tools and workflows without executing them. Which information can be used to compare and (pre-)select individual tools and entire workflows at this stage? What would we like to see in a workflow before we run it? What can (possibly) be provided by tool registries and workflow repositories?

There are many qualities and merits to consider, but there seem to be three categories of information relevant at this stage, on which we elaborate in the following: technical parameters, domain-specific considerations, and community influence (summarized in Table 1).

**Table 1.** Relevant information for static analysis of workflows.

Technical parameters	Domain-specific considerations	Community influence
• Basic tool information (such as license, version, recent updates) • (Theoretical) compatibility of tools based on their functional annotation • (Practical) compatibility of tools based on their use in existing workflows • Tool statistics like number of runs, number of users, speed, reliability, etc. • Service monitoring information about availability, uptime, downtime, runtime, etc. • Number of shims (format converters) needed in the workflow • Data format flexibility (generic vs. tailored) • Data-flow properties (such as live and dead variables) of the workflow • Control-flow properties (such as cyclomatic complexity, parallelization potential) • Tool and workflow FAIRness metrics	• Subject-specific unique or essential features of tools that the workflow needs and relation to a typical concept map in the domain • Establishment of tools (known quality metrics, well-understood configuration). Usage in commonly used workflows and known compatibilities by actual usage • Novelty of tools (new functionalities, potential for novel results, adaptation to new data types) • Similarity to existing concrete or abstract workflows (see above), workflow motifs • Type and format of produced results. Potential for direct comparison with output from other workflows • Availability of common quality control, benchmarks and benchmarking data	• Reception in the domain literature (citations, altmetrics, praise and criticism) • Reputation, someone or something being “famous” • Trends, currently popular technologies • User comments and ratings (reflecting, e.g., adequacy, understandability, usability) • Trust in developers and/or providers

*Technical Parameters*

Technical parameters of individual tools and their combinations are relevant to their operation in the context of a composite workflow. These include a whole range of properties, as indicated in Table 1. It is worth noting here that several of these properties (for static analysis at design time of the workflow) are in fact based on prior dynamic analysis of individual tools or other workflows on different levels of abstraction. This underscores the relevance of systematic dynamic analysis of scientific tools and workflows, discussed further below. For use in (automated) workflow composition, we assume the availability of such information in a tool registry like bio.tools, as additional metadata in the tool annotations. Ideally, such metadata would also be collected “by stealth” through the major community platforms, and provided to tool registries in a standard format. Similarly, an archive of historical workflow traces would be useful, which could provide representative data about prior use of tools and their combinations.

*Domain-specific considerations*

While technical parameters are important to consider for operational workflows, they need to be complemented with domain-specific considerations to obtain scientifically meaningful results. Examples of domain-specific considerations in (automated) workflow composition are given in Table 1.

*Community influence*

Finally, community dynamics also influence which tools and workflows are considered preferable. Examples are given in Table 1. While these influences are probably stronger for human workflow developers, also automated composers rely on community-provided information and are thus not agnostic to the corresponding biases.

### Dynamic analysis

Dynamic analysis refers to assessment based on execution, and naturally mostly concerns workflows at the later stages of the life cycle, when they are configured, executable and can be applied to actual data and produce results. We simply distinguish between plain executability, basic validation and systematic benchmarking in the following. Note that the focus of the discussion during the workshop was on benchmarking.

*Executability*

Executability is probably the most basic and at the same time the most important property of a computational tool or workflow. Only execution will show if the workflow actually works, if the individual tools are compatible in practice (and not only on the annotation level), and if all components have been configured correctly. In practice, it is not uncommon that this executability is applied as the only criterion: If it works and produces results at all, it is considered good enough. If it does not work, one can either try to fix it (requiring understanding why it fails) or discard it. With automated composition and workflow repositories on the rise, which give easier access to alternative workflows for the same problem, the latter is becoming an increasingly viable option.

*Validation*

Between mere executability and systematic benchmarking there is an area of workflow assessment that we here call validation. It is about critically assessing the behaviour of the workflow. Is the workflow doing what it is supposed to do? Can the results be true? Note that this notion of validation is related to, but not the same as testing, which is a separate issue for scientific software. ^100– 102^ In scientific practice, validation is often done by the workflow developer through execution of (parts of) workflows and inspection of results to see if they look as expected. Ideally, a “testing set of mind” would be taken and, for example, the workflow be tried with data that is outside of the supported range to see if/how it crashes or gives incorrect results. Such assessment can be cumbersome and challenging as it requires knowledge of both the tool functions and the applied data.

In more formal terms, we may classify these concerns into validation, verification, sensitivity analysis and uncertainty quantification (UQ) of a workflow (or workflow instance), with the canonical use of these terms being found in. ^103^ These terms, and the distinction between them, are found more often in discussions of computational models used in physics and engineering, ^104, 105^ but the concepts are relevant to (and may be adapted to) the general scientific workflows covered in this article.

In simple terms, validation asks if what we want to do is correct; verification asks if our implementation is doing what we think it is doing; and uncertainty quantification asks about how sensitive our results are, for example, to uncertainty in the initial inputs. More concretely, validation refers to the accuracy of the theoretical approach we are trying to implement (the mathematical model or analysis procedure) and is measured by agreement with 'reality' e.g. comparing predictions with experimental results. Verification is a much broader term, encompassing various assessments of the correctness of the implementation itself - is execution of my workflow or code reproducing the theoretical approach I think it is? Finally, uncertainty quantification is an enormous field in its own right, and there are many levels at which it may be introduced to a workflow, from modification of individual steps (most intrusive) through to repeatedly sampling the entire workflow as a 'black box' (least intrusive, e.g. see reference. ^106^

While only a subset of the above may be relevant to any given workflow, a scientific workflow in general will be subject to all three concerns. Adoption of automated workflow composition tools in the wider scientific community will likely come with demands for rigorous checks of correctness, and a range of definitions thereof.

*Benchmarking*

When done systematically, the dynamic analysis of computational tools and workflows is also known as benchmarking. ^59^ In the scientific workflow development life cycle ( Figure 1) benchmarking helps to determine which workflow versions or instances will be put into production for large-scale use by third parties. It assumes that the workflows are executable and validated. Generally, benchmarking can be performed with regard to scientific (e.g. analysis quality), technical (e.g. runtime performance, robustness) and usability (e.g. ease of reuse) aspects. Ideally, benchmarking uses publicly available (gold, silver, or artificial/synthetic) reference datasets. Benchmarking can be performed in a single lab (often done for benchmarking on specific aspects) or by a community of researchers (advantageous to avoid bias, often done for common and shared challenges). Due to their composite nature, workflows are generally more fragile than individual tools, which is also relevant for their benchmarking. For instance, benchmarking can help to identify problematic tools or non-interoperable tool combinations.

OpenEBench ^59^ provides a platform for community-based benchmarking of bioinformatics resources. Its scientific benchmarking component provides a virtual research environment in which individual researchers or scientific communities can share data, tools, workflows and metrics for their benchmarking challenges. The virtual research environment supports the execution of automated metrics generation workflows, and the comparison of different resources’ benchmarking results. In addition, OpenEBench has a technical monitoring component, which automatically checks basic technical properties of the registered resources, and updates the OpenEBench entry on a daily basis. Information captured here includes, for example, the availability of documentation, uptime/access time of remote resources, and the number of citations on corresponding publications. Currently metrics for the FAIR software principles ^52^ are being developed, which will also be integrated in OpenEBench’s technical monitoring component.

### Discussion of workflow assessment

Although we discussed separately static and dynamic workflow analysis, it is clear that in practice they are used together, often interleaved. Workflow developers would combine a first few tools, then execute to see what happens, and from there adapt and extend the workflow. Automated approaches to workflow development should follow this pattern, and assist the workflow developer in this incremental, checkpoint-rich style of development, rather than aiming for complete start-to-end automation. In the following we discuss three major, cross-cutting aspects of workflow assessment further: the question of what is “best”, the idea of a fitness function to measure to what extent a workflow meets its purpose, and a “great bake-off” perspective on automated composition and systematic benchmarking.

*The “best”*

**Figure 2.**
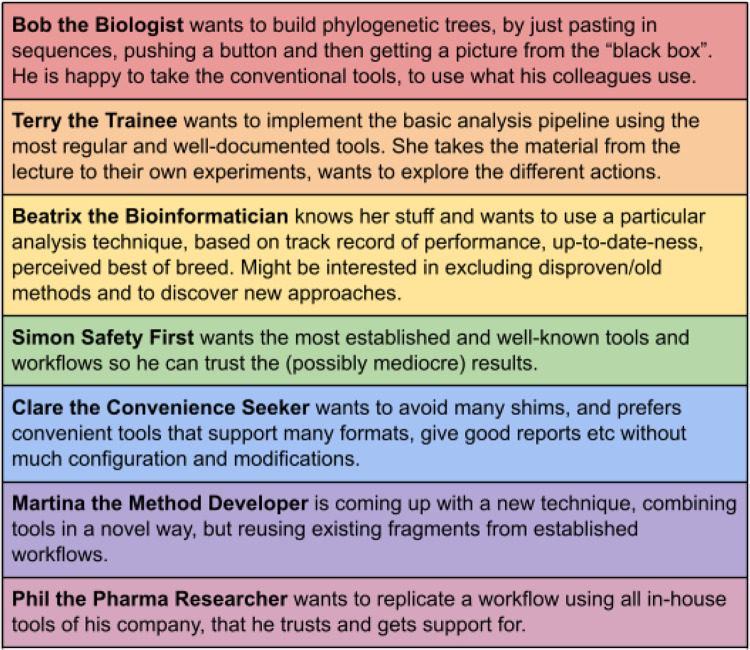
Workflow personas and use cases. Demands for configurability are ordered from low (top) to total (bottom). All participants need a publication-ready description of the provenance of their findings for perfect reproducibility. Not on the list is Rob the Routinier, who keeps doing his stuff just the way he did it for the last ten years.

Somewhat didactically, we started off the discussion about workflow assessment with the aim of looking for the “best” possible workflows. However, obviously it is highly context-dependent and often unclear what this means. With the large number of candidates potentially generated by automated composition, it is often more sensible to cull nonsensical workflows rather than trying to find the very best. Doing this with the most coarse-grained criteria will ideally lead to a significantly reduced list of options that is amenable to more fine-grained analysis and evaluation. Nevertheless, the question of what kind of workflows to prefer remains context-specific. It seems advisable to explicate concrete use cases and personas to get a better understanding of relevant situations, perspectives, and requirements. Some spontaneous ideas from the workshop are summarized in Figure 2.

Obviously these use cases and personas need to be worked out much further and only cover a part of the possible scenarios, but they clearly show that the meaning of “best” workflow spans a wide range of interpretations.

*Fitness function*

Another way to think about the problem of workflow assessment is designing a fitness function in an evolutionary computing approach or reward function in reinforcement learning. ^107^ Thus, the goal is to try to learn or optimize a particular workflow given the required output. Such an optimization lens could be useful for applying advances in machine learning or evolutionary computing to workflow composition. Thus, the aim would be to devise functions to evaluate how “fit for purpose” workflows are, given a goal like, for example, a certain benchmark. Such a function should consider functional (biological) as well as performance (computational) metrics, such as:

• Biological ground truth, what is known biologically
• Robustness to a non-specialist user, and to evolving technical requirements (execution architecture)
• Performance, reliability, reproducibility
• Tool compatibility, tool popularity
• Ratings from users who have employed workflows that are presented as possibilities (akin to looking at the ratings of a product on Amazon.com when making a purchase)
• How unique is the combination of tools.

A critical challenge for adopting this approach is the translation of the rich information both about workflows and surrounding biological knowledge into structures amenable to these optimization frameworks.

*Great Bake-Off*

We found it an appealing perspective to think about workflow assessment in the context of automated composition as a ‘Great Bake-Off’: carry out a pre-selection of automatically generated alternative workflows with low-effort assessment methods, and then let the remaining candidates compete against each other through rigorous benchmarking, in order to finally determine the workflow(s) to use in production. This process should include all areas of benchmarking (i.e., scientific, technical, and usability), though possibly with varying weighting depending on context. A similar strategy has for example successfully been taken by Automated Machine Learning (AutoML) ^35^ for the automated generation of machine learning models.

Generally, technical benchmarking is comparatively easy and objective, while scientific and usability benchmarking are quite involved, less objective and more subject to bias. They require expert knowledge and ideally many different test data sets and metrics provided by different people. Critical assessments using unpublished data with ground truth may be a reliable means to get quality benchmarking information from the communities. This can also include the creation of synthetic benchmarking data sets in case no suitable real data is available. Synthetic data might furthermore deliberately contain noise or wrong items, to test if workflows are robust or able to detect unsuitable data quality.

Obviously, for a ‘Great Bake-Off’ there needs to be a decision or agreement on the metrics used to determine the “best”. For wide acceptance, they need to be informative and domain-specific, but also community-supported. These community approaches should be objectively executed without including the tool and workflow developers. Unfortunately, such endeavours are rare at the time being.

## Roadmap

The Lorentz workshop aimed at developing a common perspective on future directions for automated composition of workflow in the life sciences. Ideas were collected in the discussion during the whole week, but the last workshop day was devoted to formulating concrete action items for the coming years. Generally speaking, the overall goal of these actions is to bring the different individual pieces discussed in the workshop together in a (more) coherent framework. We deliberately focused on actions for the next five years, acknowledging that we, in our thinking, need to distinguish between the short-term practical possibilities from long-term speculations and the things that might be achieved only over decades. In the following we outline such future directions for foundations, tooling and infrastructure, community and applications of automated workflow composition in the life sciences.

### Foundations

Achieving broad application of automated workflow composition depends on solid foundations, including a common understanding of its scope and purpose as well as community-approved definitions and standards that support these aims (see Table 2).

**Table 2.** Future work on foundations.

Action	Examples
Clarification of usage scenarios	Collect and explicate concrete user stories and scenarios, including personas (“as a <role> I want to <capability> so I can <do x>”). Elicit requirements, prioritize using the MoSCoW method.
Definition of lacking standards	Universal identifier for workflows, IDs for code and tools. Format to formally represent parameter sets in a general way. Standardized hardware constraints of software (e.g. technical parameters, firmware).
Development of lacking methods	Systematic collection and analysis of tool usage data (for funding, sustainability, benchmarking). Alignment and similarity measures between workflows, together with methods for comparing abstract and concrete workflows.
Exploration of new ideas	“Knowledge acquisition by stealth” for scaling up tool annotations and provenance trace collection. The use of workflow provenance traces for heuristic improvements of automated composition. Methods or automated workflow benchmarking, possibly reusing approaches from machine learning (AutoML).

The lack of clearly defined use cases was a recurring theme during the workshop discussions. While all approaches and methods make implicit assumptions about the usage scenarios and user groups they target, this is hardly spelled out explicitly. Their elucidation (using established methods from software requirements engineering) is a priority and will form a solid basis for future joint efforts. For example, a user who has some data and a desired endpoint, wondering how to possibly get there, might benefit from a “PipelineSketcher” that can automatically propose suitable sequences of (conceptual) operations. Another user might want to take an existing concrete workflow and get suggestions for new tools, which could be given by a “RoboAdvisor”.

Another important area of foundational work is the definition and development of lacking standards and methods, such as universal workflow identifiers and methods for meaningful workflow comparison. Furthermore, several promising, but so far little developed ideas wait to be explored further. Examples include the use of workflow provenance traces as a knowledge base for heuristic improvements of automated composition, methods for automating the benchmarking or workflows, and the concept of “knowledge acquisition by stealth”.

### Tooling and infrastructure

Developing and maintaining effective tooling and infrastructure for automated composition of workflows is hard. The workflow discussions highlighted several challenges of the current software ecosystem, in particular related to missing functionality, insufficient compatibility and interoperability, usability and convenience. Accordingly, the list of actions on tooling and infrastructure grew very easily (see Table 3).

**Table 3.** Future work on tooling and infrastructure.

Action	Examples
Provide missing functionality	Enrich bio.tools entries with additional information, e.g. annotation quality, user ratings, automated composition readiness. Enrich bio.tools with additional functionality, e.g. for finding similar tools, collection of user experience information.
Increase compatibility and interoperability	Support automated workflow composition in/to general workflow specification languages such as CWL. Exchange valid/benchmarkable workflows in common format (e.g. RO-Crate). Capture parameter settings as standardized items. Automatic conversion of data set metadata (data repositories) into correctly applied workflow parameters. Maintain dedicated libraries of helper services (shims). Improve information exchange between systems, e.g. OpenEBench, bio.tools, Conda and WorkflowHub.
Improve usability and convenience	Improve the ease and regularity of updating ontologies such as EDAM. Integrate tool recommendation, workflow exploration and user feedback features in WfMS (e.g. Galaxy), workflow repositories and registries (e.g. WorkflowHub). Use registry and workflow engine usage data for training recommendation systems. Collect tool usage data (anonymous, public) and workflow usage data (anonymous, public). Create infrastructure for (automated) workflow integration testing (in silico generated data and community-maintained test data). Support open-source community health checks (e.g. Cauldron, CHAOSS, repostatus.org).

The concept and technology of nanopublications ^108^ could possibly have a role in contributing to solutions to the above points. Nanopublications are small snippets of provenance-aware semantic representations, which can among other things be used to represent workflows, workflow steps, and the data they consume and produce. An ecosystem of tools and services around nanopublications has recently emerged that allows for decentralized and robust interaction with such semantic representations, which could now be harnessed for automated workflow composition.

Clearly, the field faces a trade-off between a limited workforce and many wishes, underscoring the need for well-defined use cases and prioritized requirements (see above) and community involvement (see below).

### Community

Community is known to be key, and not surprisingly several actions were proposed in relation to community building and involving the community in further development. Several future steps can benefit from existing communities and connect to ongoing initiatives, making them feasible in the medium term. Table 4 shows some of the concrete actions proposed.

**Table 4.** Future work on community.

Action	Examples
Community building	Use hackathons to bring the community together, e.g. propose topics in established Hackathons (e.g. BioHackathon Japan, European Biohackathon 2020). Establish a regular dedicated hackathon on the theme of automated workflow composition. Identify opportunities to train researchers to use resources and participate in the community efforts. Identify “hot topics” and forums for community mobilisation, e.g. collecting abstract workflows for an instructive “picture book of bioinformatics”.
Community development	Survey stakeholder needs, including industry, publishers (e.g. Gigascience), data repositories, frameworks (e.g. bioconductor, Linux distributions) etc. Leverage ELIXIR to drive the technical & political consolidation. Establish an ELIXIR Focus Group on automated workflow composition.

### Applications

At the beginning of the workshop several workflow applications from the fields of genomics, proteomics, proteogenomics, metabolomics, metaomics, scientometrics and text mining were presented to set the scene. After all, such applications should drive the developments, and they will mercilessly put methods, tools and infrastructure to the test. Several applications of the available workflow composition frameworks were sketched by the participants, some more domain-oriented and some more tool-oriented, but all with the potential of creating valuable insights for further developments (see Table 5).

**Table 5.** Future work on applications.

Action	Examples
Annotation of tools	Map command lines to individual tool functions. Organize available and possible shims. Annotate possible format transformations.
Automated composition of workflows	Compare the benefits of alternative methods for automated composition (exploration, recommendation, elaboration) on concrete examples. (Try to) reproduce a workflow found in a paper using literature mining and automated workflow composition.
Benchmarking of workflows	Explore the value of automated workflow composition in combination with systematic benchmarking (“Great Bake-Off”). Work towards a fully benchmarked set of >10 automatically composed proteomics workflows as a demonstrator. Collect data sets with ground truths and benchmark metrics in the omics domains.

Currently the coverage in ontologies and maturity of tool annotations vary considerably between life science domains, with genomics and more recently proteomics having received more attention. As success stories from adjacent fields are so important in encouraging joint efforts and widening adoption, we see future efforts focused on providing high-quality tool curations with concomitant ontology updates in specific fields, such as metagenomics, metaproteomics, metabolomics, epidemiology or biomedical imaging.

## Conclusion

In this report we have summarized the salient points from five days of intense scientific discourse ranging from fine-grained technical details to very broad thematic topics. Naturally, not everything that was discussed in the workshop could be included here. Despite similar ideas and efforts having struggled to find widespread application in the past, the attendees left the workshop with renewed confidence and optimism that we are at least considerably closer now, having clearly identified what development of community standards, ontologies and annotations is still needed to achieve broad adoption of automated workflow composition techniques across the life sciences.

In the time between the workshop and finalizing this report, several things have happened. For example, bio.tools received a number of new features, and continues to grow. The WorkflowHub has been released and is now in productive use. Along with this, the Bioschemas Computational Workflow Profile, ^109^ a schema.org-based specification for describing a computational workflow, has been defined. It is used by the WorkflowHub to mark up its entries and as the description of a workflow in the Workflow-RO-Crate, ^110^ the interchange packaging format which is a specialisation of the RO-Crate packaging format, ^111^ also based on schema.org. This enables workflows and associated components to be exchanged between the WorkflowHub, workflow management systems like Galaxy, Snakemake and Nextflow and their repositories, and workflow utilities like OpenEBench and LifeMonitor. Given its schema.org web markup basis, workflows marked up using the profile are readily accessible to search engines. Furthermore, case studies have been started, project proposals written, and further papers published.

While some of these developments were in some form anticipated at the workshop, others emerged from ongoing developments and urgent needs. Perhaps this is representative for a field that strives to push and challenge the frontiers of life science infrastructure. After all, the value of automated workflow composition lies in the unexpected.

## Data availability

### Underlying data

No underlying data are associated with this article.

### Extended data

Open Science Framework: Lorentz Center Workshop: Automated Workflow Composition in the Life Sciences. https://doi.org/10.17605/OSF.IO/A5EJ7. ^115^

This project contains the following extended data:

- Executive Summary.pdf (short post-workshop summary)
- Workflow Poster.jpg (workshop poster)
- Workshop Program.pdf (workshop agenda)

Data are available under the terms of the Creative Commons Attribution 4.0 International license (CC-BY 4.0).

## Footnotes

*
We use the terms “pipeline” and “workflow” interchangeably here. Another common, more differentiating view is that pipelines are purely computational and as such a subset of the more general notion of workflows, which can also involve a human element.
