# Supplementary material for: Perspectives on automated composition of workflows in the life sciences
Source: F1000Res. 2021 Sep 7;10:897. [Version 1] doi: 10.12688/f1000research.54159.1 (PMC8573700; doi:10.12688/f1000research.54159.1)
Supplement: Peer review history

# Peer review: Reviewer response for version 1

**DOI:** 10.5256/f1000research.57615.r96797

**Published:** 2021-11-05

**Authors:** Rafael Ferreira da Silva

**Affiliations:** 1 Information Sciences Institute, University of Southern California, Marina del Rey, CA, USA

This paper presents a summary of the discussions and findings from a Lorentz Center workshop organized in March 2020, with the focus on automated workflow development in the life sciences.

 Overall, the paper is well written and presents a community view on the current state-of-the-art, challenges, and perspectives for research efforts. Another key contribution of the paper is the development of a schematic process of the scientific workflow life cycle, which is comprised of six principal stages, each with their associated artifacts. Although a survey of state-of-the-art technologies is not the goal of the paper, it still provides a reasonable amount of background information.

 On the downside, the paper could better articulate which properties are inherent to each of the aforementioned stages, and organize the discussions around them.

 Specific comments:

- I would recommend authors to identify common properties for the set of tools described in the paper. The current text alludes to individual tool functionalities but does not clearly discuss their common ground / specificities.
- It is not clear to me how "workflow benchmarks" are defined. At some moments I see them as "reference workflows" for validating the workflow structure and data, while at other sections I see them as performance drivers. It might be good to provide an actual definition of the term, or even separate them (as they seem to measure different properties).
- The proposed future work (foundations, tooling and infrastructure, community, and applications) could leverage several ongoing efforts from the scientific workflows community such as in [1, 2, 3].

Is the topic of the opinion article discussed accurately in the context of the current literature?

Yes

Are arguments sufficiently supported by evidence from the published literature?

Yes

Are all factual statements correct and adequately supported by citations?

Yes

Are the conclusions drawn balanced and justified on the basis of the presented arguments?

Yes

Reviewer Expertise:

scientific workflows, distributed computing, modeling and simulation of distributed systems, parallel computing

I confirm that I have read this submission and believe that I have an appropriate level of expertise to confirm that it is of an acceptable scientific standard.

# Peer review: Reviewer response for version 1

**DOI:** 10.5256/f1000research.57615.r94267

**Published:** 2021-10-19

**Authors:** Pinar Alper

**Affiliations:** 1 Luxembourg Centre for Systems Biomedicine, University of Luxembourg, Belvaux, Luxembourg

The paper is the final report from a 2020 workshop on automated workflow development in life sciences.

 This is overall a well written paper, which, scope-wise, is framed by the discussions of the workshop rather than being an exhaustive state-of-the-art paper on semantic workflow development techniques. The paper is lengthy and covers many topics in a partly explored research space in scientific workflows, it requires multiple reads to have an overall viewpoint.

**The contributions of the paper are the following:**

- A scientific workflow lifecycle outlining the various activities and artefacts in stages of the lifecycle. I think this is the strongest contribution of the paper. It acts as a map for authors to place various past and future research.
- A survey of current technologies and practices that directly or indirectly support workflow composition. This is not an exhaustive survey reporting research and techniques in workflow composition, instead focuses on what is currently working and available to life science researchers.
- A set of future directions on research and technology development to enable automated workflow composition across the lifecycle.

The paper reports on a stimulating topic and can act a kick starter for the next era of research on the intersection of semantic technologies and scientific workflows. 

**Suggestions for improvements:**

- The paper does not utilise section numbers which makes reading difficult. If the journal format allows I’d recommend the use of numbering.
- Abstract reads “Recent technological advances have returned …” What are those? Give one example in the abstract.
- In the introduction you do not mention explicitly the bioinformatician but say “To biologists there is a latent fear to have chosen …” and later mention “human expert knowledge” which I guess refers to the bioinformatician. So I gather automated composition will ultimately assist the bioinformatician. Without such clarification the paragraph reads as if biologists due to their “latent fear of making a mistake” will consult “automated assembly”. I believe that is an overly optimistic statement without any reference to any surveys or observations on biologists’ and bioinformaticians’ expectations or experience with workflows. During peer-review scientists are held _accountable_ for their analytical methods and in this paragraph it is not justified with any references how an automated decision made by composition software increases trustworthiness of the method.
- The introduction states the ”developments bring the long standing vision of automated composition … within reach”. Here 1-2 sentences, will be helpful, that summarise to date how much progress has been made on this vision (a very brief summary of page 11 essentially).
- The paper often cites multiple papers to refer to the same system or approach. Taverna, Galaxy, myExperiment, Biocatalogue, Ontosoft, Magallenes, Prophets are all cited with refs to multiple papers. While this is useful to have an overview of all papers, it would help the reader if you cited the definitive or the more relevant papers for these systems. If both citations are needed then footnotes will be helpful on what different can be found in each particular paper for one system. Tightening up the citations could also help creating space for citations of past workflow systems which have addressed assisted workflow composition e.g. Vistrails or perhaps references to any useful reviews (if exist) on the topic.
- Static analysis of scientific workflows* (an area that has received limited attention), is closely tied to data and control flow constructs in workflow languages and provenance. Static analysis (re: technical parameters) can tell whether resulting provenance traces from a workflow would provide granular traceability or not. This may as well be another technical parameter aiding automated composition.
- Static analysis over workflows re: domain-specific characteristics has so far been done to understand, compare and contrast various scientific domains and their use of workflows **. Findings from such studies may help in refine “domain-specific considerations”.

* Alper P, Belhajjame K, Goble C: Static analysis of Taverna workflows to predict provenance patterns. *Future Generation Computer Systems*. 2017; **75**: 310-329

 ** Garijo D, Alper P, Belhajjame K, Corcho O, et al.: Common motifs in scientific workflows: An empirical analysis. *Future Generation Computer Systems*. 2014; **36**: 338-351

Is the topic of the opinion article discussed accurately in the context of the current literature?

Yes

Are arguments sufficiently supported by evidence from the published literature?

Yes

Are all factual statements correct and adequately supported by citations?

Yes

Are the conclusions drawn balanced and justified on the basis of the presented arguments?

Yes

Reviewer Expertise:

scientific workflows, provenance, data protection

I confirm that I have read this submission and believe that I have an appropriate level of expertise to confirm that it is of an acceptable scientific standard.

## References

[1] : Static analysis of Taverna workflows to predict provenance patterns. *Future Generation Computer Systems* .2017;75: 10.1016/j.future.2017.01.004 310-329 10.1016/j.future.2017.01.004

[2] : Common motifs in scientific workflows: An empirical analysis. *Future Generation Computer Systems* .2014;36: 10.1016/j.future.2013.09.018 338-351 10.1016/j.future.2013.09.018
